# Accuracy of Ultrasound Diagnosis of Thyroid Nodules Based on Artificial Intelligence-Assisted Diagnostic Technology: A Systematic Review and Meta-Analysis

**DOI:** 10.1155/2022/9492056

**Published:** 2022-09-23

**Authors:** Yu Xue, Ying Zhou, Tingrui Wang, Huijuan Chen, Lingling Wu, Huayun Ling, Hong Wang, Lijuan Qiu, Dongqing Ye, Bin Wang

**Affiliations:** ^1^Department of Epidemiology and Biostatistics, School of Public Health, Anhui Medical University, Hefei, Anhui, China; ^2^Inflammation and Immune Mediated Diseases Laboratory of Anhui Province, Hefei, Anhui, China

## Abstract

**Background:**

Ultrasonography (US) is the most common method of identifying thyroid nodules, but US images require an experienced surgeon for identification. Many artificial intelligence (AI) techniques such as computer-aided diagnostic systems (CAD), deep learning (DL), and machine learning (ML) have been used to assist in the diagnosis of thyroid nodules, but whether AI techniques can improve the diagnostic accuracy of thyroid nodules still needs to be explored.

**Objective:**

To clarify the accuracy of AI-based thyroid nodule US images for differentiating benign and malignant thyroid nodules.

**Methods:**

A search strategy of “subject terms + key words” was used to search PubMed, Cochrane Library, Embase, Web of Science, China Biology Medicine (CBM), and China National Knowledge Infrastructure (CNKI) for studies on AI-assisted diagnosis of thyroid nodules based on US images. The summarized receiver operating characteristic (SROC) curve and the pooled sensitivity and specificity were used to assess the performance of the diagnostic tests. The quality assessment of diagnostics accuracy studies-2 (QUADAS-2) tool was used to assess the methodological quality of the included studies. The Review Manager 5.3 and Stata 15 were used to process the data. Subgroup analysis was based on the integrity of data collection.

**Results:**

A total of 25 studies with 17,429 US images of thyroid nodules were included. AI-assisted diagnostic techniques had better diagnostic efficacy in the diagnosis of benign and malignant thyroid nodules: sensitivity 0.88 (95% CI: (0.85–0.90)), specificity 0.81 (95% CI: 0.74–0.86), diagnostic odds ratio (DOR) 30 (95% CI: 19–46). The SROC curve indicated that the area under the curve (AUC) was 0.92 (95% CI: 0.89–0.94). Threshold effect analysis showed a Spearman correlation coefficient: 0.17 < 0.5, suggesting no threshold effect for the included studies. After a meta-regression analysis of 4 different subgroups, the results showed a statistically significant effect of mean age ≥50 years on heterogeneity. Compared with studies with an average age of ≥50 years, AI-assisted diagnostic techniques had higher diagnostic performance in studies with an average age of <50 years (0.89 (95% CI: 0.87–0.92) vs. 0.80 (95% CI: 0.73–0.88)), (0.83 (95% CI: 0.77–0.88) vs. 0.73 (95% CI: 0.60–0.87)).

**Conclusions:**

AI-assisted diagnostic techniques had good diagnostic efficacy for thyroid nodules. For the diagnosis of <50 year olds, AI-assisted diagnostic technology was more effective in diagnosis.

## 1. Introduction

Thyroid nodules (TN) are lumps in the thyroid gland that move up and down with the thyroid gland with swallowing movements and are a common clinical condition that can be caused by a variety of causes [1]. There are also a variety of disease types that may occur in clinical TN, for example, thyroid degeneration, inflammation, autoimmunity, and new organisms can all appear in the form of nodules [2, 3]. It can be single or multiple. Multiple nodules have a higher incidence than individual nodules, but single nodules account for a greater proportion of thyroid cancer [4]. Furthermore, TN are common in iodine-deficient areas, in women, and patients receiving neck irradiation [5]. High-definition thyroid ultrasonography (US) is the most sensitive method for evaluating TN [6]. It can determine the nature of TN, such as the location, morphology, size, number, nodule marginal state, internal structure, echo form, blood flow status, and cervical lymph node conditions. However, the diagnostic performance of the US depends heavily on the clinical experience of radiologists, and secondly, the data generated by US examination is large and complex, the workload of manual analysis is huge, and it is susceptible to environmental, instrumental, and human subjective factors [7–10].

Artificial intelligence (AI) is the science of applying intelligent machines and systems to mimic the ability of human intelligent activity, and image recognition using AI methods is one of the most developed branches of AI. Many AI-assisted diagnostic techniques have been widely used for the differential diagnosis of TN. With the advent of various TI-RADS, the goal of applying AI to TN reflects the goal of TI-RADS: to improve reporting consistency and enhance diagnostic performance [11]. Commonly used AI-aided diagnostic techniques include computer-aided diagnostic systems (CAD), machine learning (ML), deep learning (DL), and so on [12]. AI processes a variety of holographic information in parallel to easily identify and simulate complex nonlinear relationships in images [13, 14]. In addition, AI can extract and quantify critical image information, transforming image diagnostics from subjective qualitative tasks into objective quantitative analysis. Based on this, the combination of AI and medical big data has led to a new diagnostic method, namely, CAD technology. On the one hand, the application of objective and stable, easy to operate, high accuracy CAD software helps to speed up the diagnosis and treatment process of US doctors and shorten the waiting time; on the other hand, it improves the accuracy and consistency of TI-RADS classification and avoids excessive fine-needle aspiration (FNA) caused by subjective factors and diagnostic techniques. Since the initial reporting of the diagnostic performance of the CAD system for thyroid lesions [15], several studies have shown that CAD methods have improved the diagnostic performance of thyroid US [16, 17].

In addition, there have been many studies through meta-analysis CAD systems for the diagnosis of TN which had high efficacy [18–20], but there was no meta-analysis for AI-assisted diagnostic technology, this system not only includes studies containing CAD systems but also includes some ML and DL AI models for systematic review and meta-analysis, aiming to evaluate the accuracy of AI-assisted diagnostic techniques in diagnosing malignant TN.

## 2. Methods

### 2.1. Search Strategy and Selection Criteria

PubMed, Cochrane Library, Embase, Web of Science, China Biology Medicine (CBM), and China National Knowledge Infrastructure (CNKI) databases were searched until April 27, 2022. A study evaluated the performance of AI-assisted diagnostic techniques in distinguishing malignant from benign TN on US. There was no limit to the kinds of languages published. The following thematic terms were used: “ultrasonography,” “diagnostic ultrasound,” and “ultrasound imaging;” “thyroid nodules,” “thyroid gland,” and “thyroid cancer;” “artificial intelligence,” “deep learning,” “computer-assisted,” “machine learning,” and “neural network.”

The inclusion criteria were as follows: (1) patients with TN were diagnosed by high-definition thyroid US; (2) FNA or surgical biopsy as the “gold standard” for diagnosing malignant TN; (3) sensitivity and specificity should be provided. These studies were excluded: (1) the article types were reviewed, including animal experiments and meta-analysis; (2) the full text was not obtained; (3) the data provided were incomplete.

When multiple algorithms are involved, only the one with the highest accuracy or the largest area under the curve (AUC) was selected for analysis. When evaluating the performance of AI-assisted diagnostics through multiple external validation groups, only the largest cohort was selected for analysis.

### 2.2. Data Extraction and Quality Assessment

The two researchers (Yu Xue and Ying Zhou) independently completed the selection process and resolved their differences through discussion. Two other researchers (Tingrui Wang and Huijuan Chen) independently extracted detailed data from the identified literature and cross-examined them to ensure that the information collected was accurate. Any disputes in the extraction or evaluation of the data were resolved through panel discussion or arbitration by the head of the study. The specific information extracted from each study included the following: first author, study country, publication year, study design, study methodology, number of cases, number of US images, age, sex, nodule diameter, sensitivity, and specificity. The methodological quality of each study was assessed using the quality assessment of diagnostics accuracy studies-2 (QUADAS-2) tool [21].

### 2.3. Statistical Analysis

Statistical analysis was performed using Review Manager 5.3 and STATA 15.0 software. By constructing the hierarchically summarized ROC (SROC) curve, the diagnostic efficacy was evaluated by pooled sensitivity, specificity, diagnostic odds ratio (DOR), and AUC of 95% CI. The DOR reflects the degree to which the results of a diagnostic test are linked to the disease. When the DOR value >1, the larger the value, the better the discrimination effect of the diagnostic test; when the value <1, normal people are more likely to be judged positively by the diagnostic test than the patient; when the value = 1, it means that the diagnostic test cannot distinguish between normal people and patients.

Publication bias was assessed using Deek's funnel chart asymmetry test. The Spearman correlation coefficient was used to evaluate threshold effects between studies. Heterogeneity assessment was performed by Cochran's *Q*-test and the I-square (*I*^2^) statistic. When the *I*^2^ ≥ 50%, the *p* value of the Cochran *Q* test was less than 0.1, the results showed that there was heterogeneity in the results, meta-regression was used to find the source of heterogeneity, and subgroup analysis was performed on the variables that produced heterogeneity. A *p*-value <0.05 was considered statistically significant.

## 3. Results

### 3.1. Literature Search and Study Characteristics

**Figure 1:**
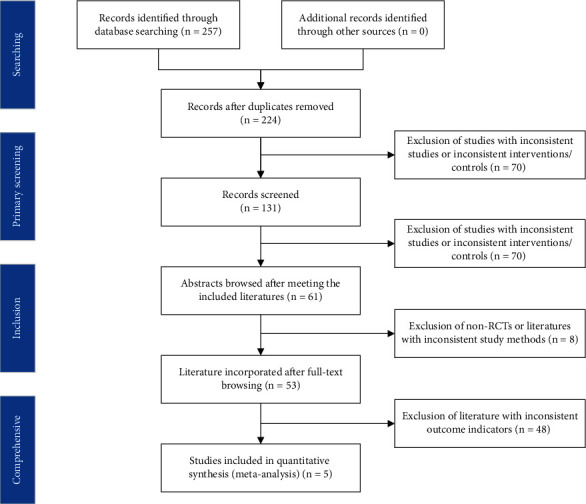
PRISMA diagram for the systematic review.

After a comprehensive computer search and extensive cross-checking of reference lists, a total of 162 records were obtained. 27 studies were excluded for repetitive reasons. 21 reviews were removed. After a comprehensive review of the remaining 114 studies, a total of 25 studies that met the selection criteria were eventually included in the meta-analysis. This meta-analysis was planned and performed following the Preferred Reporting Items for Systematic Reviews and Meta-Analysis Guideline (Figure 1).

Of the 25 included studies, 7 were prospective (28%) and 18 retrospective studies (72%); 12 were conducted in CAD (48%), 13 were conducted in DL or ML (52%); 13 studies were from China (52%), 8 studies from Korea (32%), Australia (4%), Poland (4%), Italy (4%) and the United States (4%) each had 1 study. In addition, the average age of participants in 19 studies were <50 years (76%), and the average age of participants in 6 studies were ≥50 years (24%). A total of 17 429 US images of TN were included (Table1).

### 3.2. Study Quality Assessment

**Figure 2:**
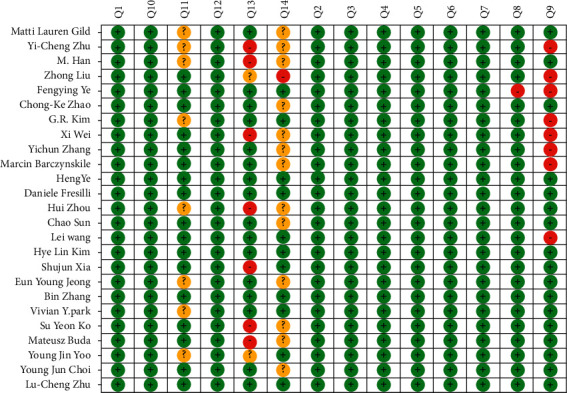
Methodological quality of the included studies: the summary of risk of bias and applicability concerns for the included studies.

**Figure 3:**
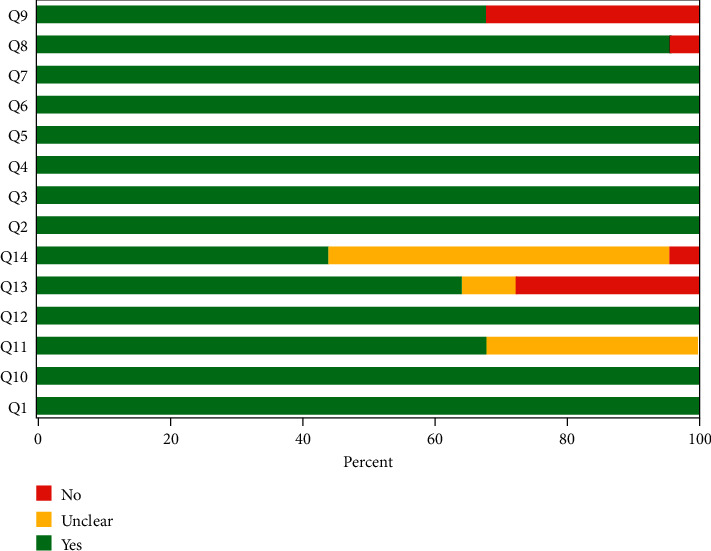
Methodological quality of the included studies: the quality of individual studies.

The evaluation results for QUADAS-2 indicated that the overall quality of the studies included in the analysis ranged from medium to high (Figures 2 and 3). The quality of the included studies was considered satisfactory.

### 3.3. Diagnostic Accuracy and Heterogeneity Evaluation

**Figure 4:**
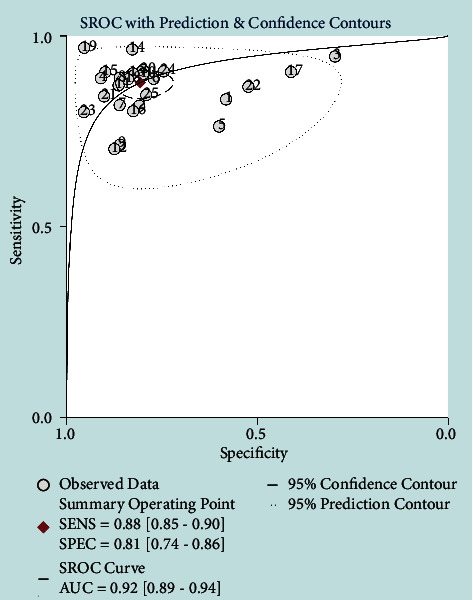
Summary receiver operating characteristic (SROC) curves of AI-aided diagnostic techniques for the diagnosis of TN.

**Figure 5:**
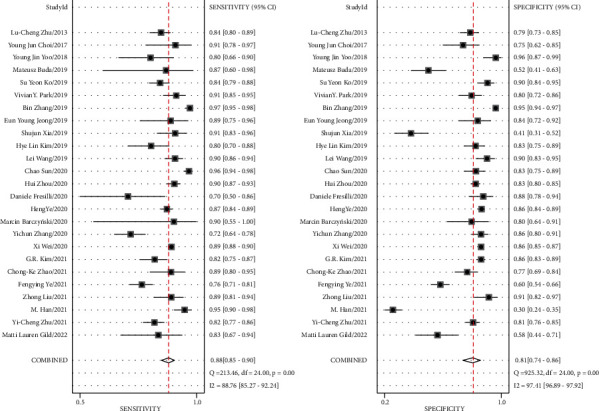
Forest plot of the comprehensive sensitivity and specificity of AI-aided diagnostic techniques for diagnosing TN.

Of the 25 studies included in the meta-analysis, the results of the diagnostic performance of AI-assisted diagnostic techniques on TN showed that pooled sensitivity, specificity, positive likelihood ratio (PLR), negative likelihood ratio (NLR) and DOR were 0.88 (95% CI: 0.85–0.90), 0.81 (95% CI: 0.74–0.86), 4.5 (95% CI: 3.4–6.1), 0.15 (95% CI: 0.12–0.19) and 30 (95% CI: (19–46)) (Table 2). The SROC curve showed that the AUC was 0.92 (95% CI: (0.89–0.94)) (Figure 4). Although these results indicated that AI-assisted diagnostic techniques had good diagnostic efficacy for TN, there was a high heterogeneity when analyzing the pooled sensitivity and specificity. The pooled sensitivity and specific heterogeneity of AI-assisted diagnostic techniques in meta-analysis were *I*^2^ = 88.75% (95% CI: 85.26%–92.24%) and *I*^2^ = 97.41% (95% CI: 96.89%–97.92%) (Figure 5). To explore the sources of heterogeneity, we analyzed the effect of threshold effects.

**Figure 6:**
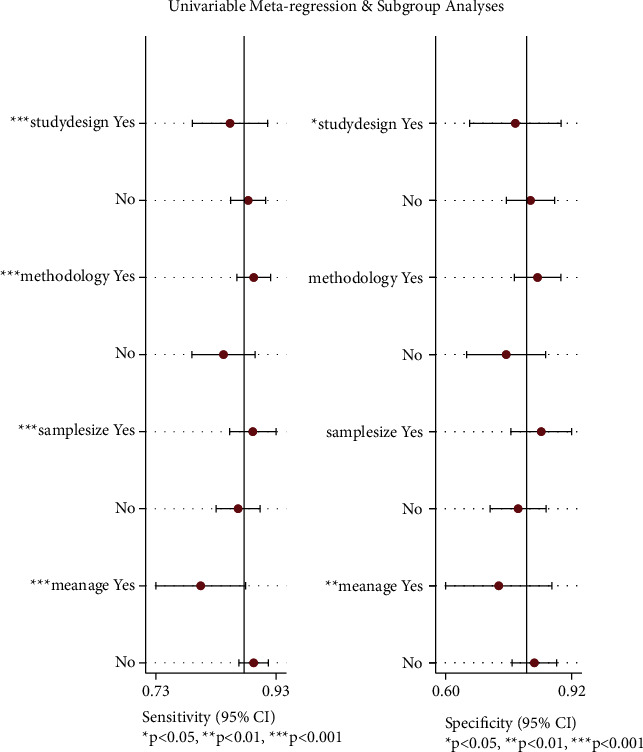
Meta-regression analysis of different study designs, methodologies, sample sizes and mean ages.

The effect of threshold effects on heterogeneity was assessed by calculating the Spearman correlation coefficient. The result showed that the Spearman correlation coefficient was 0.17 (*p*=0.418), and indicated that there was no threshold effect. After excluding the effects of threshold effects on heterogeneity, based on the completeness of the collected data, we performed analyses of 4 subgroups (study design, methodology, sample size and mean age) to determine the source of heterogeneity. The study design was divided into prospective and retrospective studies, with research methods divided into CAD and DL (including ML), sample sizes were divided into ≥500 and <500, and the average age was divided into ≥50 years and <50 years. Meta-regression analysis of 4 subgroups found that the effects of 4 subgroups on sensitivity heterogeneity were statistically significant, the effect of study methods and sample size on specific heterogeneity was not statistically significant (Figure 6), and the results of the combined model showed that only the mean age subgroup had statistical significance for sensitivity and specificity (Table 3). AI-assisted diagnostic systems had high sensitivity and specificity for people aged <50 years (0.89 (95% CI: 0.87–0.92) vs. 0.80 (95% CI: 0.73–0.88)), (0.83 (95% CI: 0.77–0.88) vs. 0.73 (95% CI: 0.60–0.87)).

**Figure 7:**
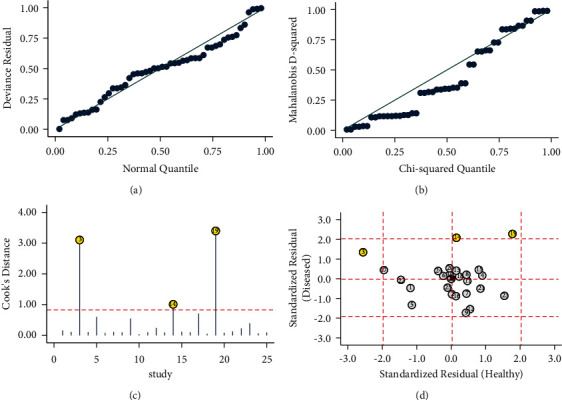
Sensitivity analysis of AI-assisted diagnostic technique for TN diagnosis. (a) Graphical depiction of residual-based goodness-of-fit; (b) Bivariate normality; (c) influence; (d) outlier detection.

Considering the large number of included studies, there may be some other factors that have an impact on the combined results, so we conducted a further sensitivity analysis (Figure 7). The goodness of fit (a) and bivariate normality (b) show the degree of fitting of the regression line to the observed value. As shown, the observed value was distributed around the reference line. The observed values were stable. The influence analysis (c) indicated that 3 studies may overestimate the pooled results. The outlier detection test indicated (d) that 3 studies were out of the detection range. After excluding these studies, the pooled specificity did not change. The pooled sensitivity changed from 0.88 (95% CI: 0.85–0.90) to 0.85 (95% CI: 0.83–0.88) without significant changes. The sensitivity analysis results showed that the meta-analysis had good robustness.

**Figure 8:**
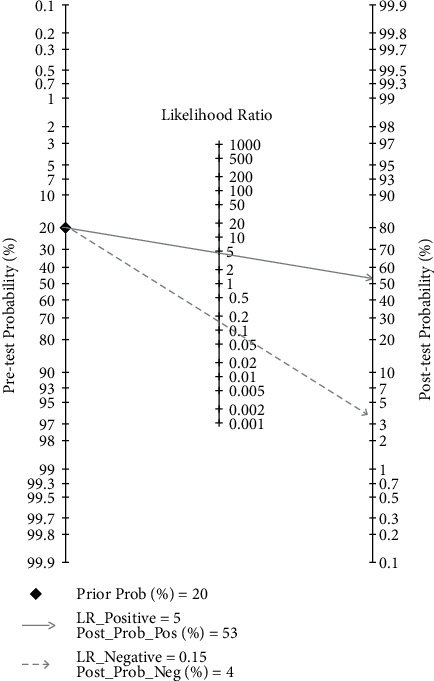
Evaluation of clinical applicability of AI-assisted diagnostic techniques in TN diagnosis: Fagan nomogram.

**Figure 9:**
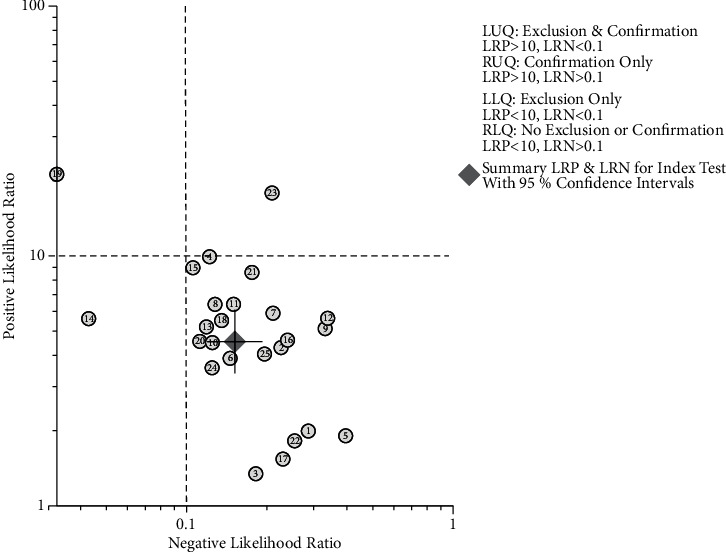
Evaluation of clinical applicability of AI-assisted diagnostic techniques in TN diagnosis: likelihood ratio scattergram.

**Figure 10:**
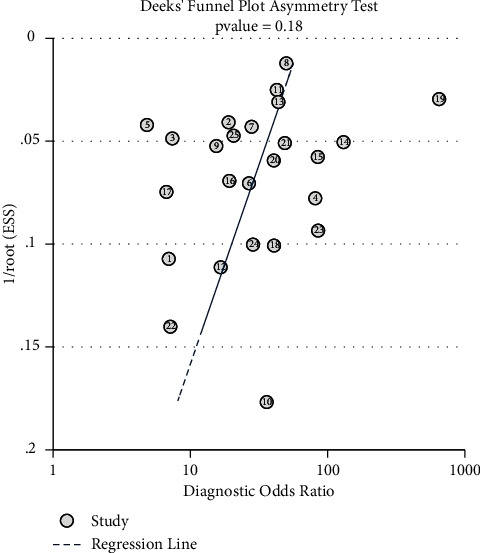
Results of Deeks' funnel plot of asymmetry test for publication bias.

Assessment of the clinical applicability of AI-assisted diagnostic techniques for diagnosis on TN founded that when the pretest probability was set at 20%, the post‐test probability for a positive test result was 53%. When the NLR was set at 0.16, the post‐test probability reduced to 4% for a negative test result (Figure 8). The diagnostic performance was visualized by the likelihood ratio scattergram and PLR > 10 and NLR < 0.1 represented a high diagnostic accuracy (Figure 9). The Deek's funnel asymmetry test showed *p* = 0.18 with no publication bias (Figure 10). All of these results suggested that the degree of diagnostic accuracy of AI-assisted diagnostic techniques for detecting malignant TN was relatively high. Figure 1 showed the flow chart of our literature search and screening based on the PRISMA statement of systematic reviews. Figures 2 and 3 showed the methodological quality assessment of all the included studies.  Figure 2 shows the overall quality assessment, and Figure 3 showed the quality assessment of each study. Figure 4 shows the summary receiver operating characteristic (SROC) curves of AI-aided diagnostic techniques for the diagnosis of TN. Figure 5 showed the forest plot of the comprehensive sensitivity and specificity of AI-aided diagnostic techniques for diagnosing TN. Figure 6 showed the results of the meta-regression analysis for different subgroups. Figures 8 and 9 showed the results of evaluation of clinical applicability of AI-assisted diagnostic techniques in TN diagnosis. Figure 10 showed the assessment of publication bias for all studies included in the analysis

## 4. Discussion

As the main method for diagnosing TN, the US has the characteristics of easy operation, affordable price and no radioactivity hazards [47]. The US also is an important means of helping radiologists assist in diagnosing TN [48]. At the same time, the accuracy and reliability of the diagnosis depend on the quality of the image and the expertise of the radiologist, so there is a certain instability and inaccuracy in the diagnosis based on the radiologist [49]. In addition, different types of TN have different features in the US images, further affecting the accuracy of the radiologist's diagnosis [50]. FNA and pathological biopsy are currently the “gold standard” for identifying malignant TN, but compared to the US, FNA and pathological biopsies are not only expensive but also have some damage to the patient itself [51]. The application of AI in imaging provides good help for the diagnosis of malignant TN based on the US, and many AI-assisted diagnostic techniques have been used to diagnose TN, but the accuracy of these techniques varies greatly [52–54].

AI is a technology used to extract and quantify key image information by simulating complex human functions and can extract and quantify key image information, whereby image diagnosis converts from a subjective qualitative task to objective quantitative analysis [51]. This more detailed and precise information is conducive to special risk stratification and propels tailored management to transit from the surface (population-based) to a point (individual-based) [14, 55]. Interestingly, the AI model appears to be a promising tool to facilitate a better knowledge of TN via quantitative analysis of typical US features and introduction of texture features. In this meta-analysis, we were the first to summarize studies on the diagnostic accuracy of the AI-assisted diagnostic systerms based on US images for TN, and 25 studies from 6 different countries were included in the pooled analysis. In all the studies included in the analysis, it was suggested that the AI-assisted diagnostic systems had a good diagnostic efficiency for TN. The results of pooled analysis showed that the pooled sensitivity, specificity, PLR, NLR and DOR were 0.88 (95% CI: 0.85–0.90), 0.81 (95% CI: 0.74–0.86) and 4.5 (95% CI: 3.4–6.1), 0.15 (95% CI: 0.12–0.19) and 30 (95% CI: 19–46), and the AUC of SROC curve was 0.92 (95% CI: 0.89–0.94). The heterogeneity of sensitivity and specificity between studies was high. First, considering the effect of the threshold effect on meta-analysis heterogeneity in diagnostic experiments, the effect of the threshold effect was analyzed using the Spearman correlation coefficient, and the result showed that there was no threshold effect; then, due to the completeness of the included study data, the meta-regression analysis of 4 different subgroups was performed in this study and the effect of whether the mean age ≥50 years on heterogeneity was statistically significant. In addition, this study also found that AI-assisted diagnostic technology was less effective in diagnosing the ≥50-year-old age group than the <50-year-old age group. Advanced age is a risk factor for the development of TN [56–58]. For TN with complex characteristics, not only the diagnostic effect of AI-assisted diagnosis technology was poor, but also the diagnostic level of radiologists was relatively low.

Although there is evidence that CAD systems and some ML and DL models in AI-assisted diagnostic techniques can improve the accuracy of malignant TN [59–63], the evidence is limited and there has been no systematic evaluation. Compared with the previous research, some ML, DL models and CAD systems that assist diagnosis are uniformly classified into AI-assisted diagnostic technologies, and the diagnostic efficacy is meta-analyzed. This study found that AI-assisted diagnostic techniques have high sensitivity and specificity, which is consistent with the results of Zhao WJ et al. to evaluate the diagnostic efficacy of CAD systems [64, 65]. At the same time, this study also found that whether this prospective study has a certain impact on the diagnostic efficacy of AI-assisted diagnostic technology, and retrospective studies have better sensitivity and specificity than prospective studies. In addition, Xu et al. evaluated caddy systems to meta-analyze the diagnosis of TN and found that the CAD system was more effective in diagnosis, but experienced radiologists may still have advantages over CAD systems during real-time diagnosis [66]. Combined with the results of this study, AI-assisted diagnostic technology still needs to be improved for prospective and real-time diagnosis of TN.

This study also had some limitations. First, various AI models were incorporated in this meta-analysis, which may introduce statistical heterogeneity. Secondly, because some basic features of TN, such as nodule diameter, echo form, and internal structure, were not included, the influence of these basic features of heterogeneous sources on diagnostic efficacy cannot be further explored. Finally, different types of TI-RADS, such as ATA-TIRADS, ACR-TIRADS, and K-TIRADS, were included in this study, but some studies did not indicate specific TI-RADS, no further analysis of different types of TI-RADS was carried out.

In summary, this meta-analysis investigated the diagnostic efficacy of AI-assisted diagnostic technology based on the US images on TN, including different ML, DL models and CAD systems, and it had good diagnostic efficacy. For the diagnosis of <50 year olds, AI-assisted diagnostic technology was more effective in diagnosis. Given the limitations of this analysis, further research is needed to explore better AI-assisted diagnostic techniques.

## Figures and Tables

**Table 1:** Basic characteristics of included studies.

References	Year	Country	Design	Methodology	Sample size	Mean age	B	M	Sen	Spec
Gild et al. [22]	2022	Australia	R	ML	91	60.10	55	36	0.82	0.59
Zhu et al. [23]	2021	China	R	DL	600	55.20	300	300	0.82	0.81
Han et al. [24]	2021	Korea	R	CAD	454	49.50	287	167	0.95	0.30
Zhong Liu [25]	2021	China	R	DL	175	44.34	67	96	0.89	0.91
Fengying Ye [26]	2021	China	P	CAD	565	54.10	270	295	0.76	0.60
Chong-Ke Zhao [27]	2021	China	R	ML	223	48.85	136	80	0.89	0.77
G.R. Kim [28]	2021	Korea	P	DL	760	51.00	584	176	0.82	0.86
Xi Wei [29]	2020	China	R	DL	7 216	45.29	2712	4504	0.89	0.86
Yichun Zhang [30]	2020	China	R	CAD	365	46.40	179	186	0.72	0.86
Marcin Barczyński [31]	2020	Poland	P	CAD	50	47.50	40	10	0.90	0.80
Heng Ye [32]	2020	China	R	DL	1 601	45.16	861	740	0.87	0.86
Daniele Fresilli [33]	2020	Italy	R	CAD	107	55.00	80	27	0.70	0.88
Hui Zhou [34]	2020	China	R	DL	1097	47.30	669	428	0.90	0.83
Chao Sun [35]	2020	China	R	DL	550	43.00	128	422	0.96	0.83
Lei Wang [36]	2019	China	R	DL	351	45.76	109	242	0.91	0.90
Hye Lin Kim [37]	2019	Korea	R	CAD	218	48.00	132	86	0.80	0.83
Xia et al. [38]	2019	China	P	CAD	180	47.20	85	95	0.91	0.41
Jeong et al. [39]	2019	Korea	P	CAD	100	46.00	56	44	0.89	0.84
Zhang et al. [40]	2019	China	R	ML	1 238	45.25	788	450	0.97	0.95
Park et al. [41]	2019	Korea	R	DL	286	47.18	130	156	0.91	0.80
Ko et al. [42]	2019	Korea	R	DL	439	46.70	143	296	0.84	0.90
Buda et al. [43]	2019	USA	R	DL	99	52.20	84	15	0.87	0.52
Yoo et al. [44]	2018	Korea	P	CAD	117	43.20	67	50	0.80	0.96
Choi et al. [45]	2017	Korea	P	CAD	102	45.30	59	43	0.91	0.75
Zhu et al. [46]	2013	China	R	DL	464	47.70	187	277	0.85	0.79

P, prospective; R, retrospective; B, benign; M, malignant; Sen, sensitivity; Spec, specificity.

**Table 2:** Summary performance estimates.

Parameter	Estimates	95% CI
Sensitivity	0.88	0.85–0.90
Specificity	0.81	0.74–0.86
PLR	4.5	3.4–6.1
NLR	0.15	0.12–0.19
DOR	30	19–46

PLR, positive likelihood ratio; NLR, negative likelihood ratio; DOR, diagnostic odds ratio.

**Table 3:** Meta-regression for heterogeneity within studies.

Parameter	Number of studies	Sensitivity estimates (95% CI)	*p* value	Specificity estimates (95% CI)	*p* value	*I * ^2^ in joint model estimates (95% CI)	*p* value
Design							
P	7	0.85 (0.79–0.92)	<0.001	0.78 (0.66–0.90)	0.02	0% (0%–100%)	0.55
R	18	0.88 (0.85–0.91)		0.82 (0.75–0.88)			

Methodology							
DL	15	0.89 (0.87–0.92)	<0.001	0.84 (0.77–0.90)	0.14	60% (11%–100%)	0.08
CAD	10	0.84 (0.79–0.90)		0.75 (0.65–0.86)			

Sample size							
≥500	8	0.89 (0.85–0.93)	<0.001	0.84 (0.77–0.92)	0.09	0% (0%–100%)	0.38
<500	17	0.87 (0.83–0.90)		0.78 (0.71–0.86)			

Mean age							
≥50	6	0.80 (0.73–0.88)	<0.001	0.73 (0.60–0.87)	0.01	75% (46%–100%)	0.02
<50	19	0.89 (0.87–0.92)		0.83 (0.77–0.88)			

P, prospective; R, retrospective; DL, deep learning and machine learning; CAD, computer-aided diagnostic systems.

## Data Availability

The data supporting this meta-analysis were from previously reported studies and datasets, which have been cited. The processed data are available from the corresponding author upon request.

## Abbreviations

AI: Artificial intelligence
CAD: Computer-aided diagnostic systems
DL: Deep learning
ML: Machine learning
US: Ultrasonography
AUC: Area under the receiver operating characteristic
CI: Confidence interval
DOR: Diagnostic odds ratio
PLR: Positive likelihood ratio
NLR: Negative likelihood ratio
QUADAS-2: Quality assessment of diagnostics accuracy studies-2
SROC: Summary receiver operating characteristic
FNA: Fine-needle aspiration
TI-RADS: Thyroid imaging reporting and data system.

## Supplementary Materials

Supplementary Materials Table S1. Search strategies. Table S2. QUADAS-2.
